# Evaluating trauma care: comparison of early versus late tracheostomy ICU data outcome on injured patients

**DOI:** 10.1186/cc14397

**Published:** 2015-03-16

**Authors:** V Kaldis, N Mourelatos, D Markopoulou, K Venetsanou, E Diogou, E Papadaki, D Chroni, I Alamanos

**Affiliations:** 1 General Hospital KAT-EKA, Kifisia Athens, Greece; 2 ICU Research Unit KAT, Kifissia Athens, Greece

## Introduction

In the surgical ICU, bedside tracheostomy (T) is one of the most frequently applied surgical techniques for multi-injured patients mainly with TBI [1]. The optimum surgical time decision for T still remains a contradiction in trauma. This retrospective study was designed to register all trauma patients who underwent T, during 60 months of observation (2009 to 2013), in order to identify factors associated with their ICU outcome on the basis of the T day (A <10th day >B) after tracheal intubation.

## Methods

Seventy-eight injured patients in the SICU underwent T, from a total of 403 issues; 58 male and 20 female, with mean age 59.3 and 74.7 years respectively. The total length of ICU stay recorded was 2,098 days, nursing time 26.55 (4/93), whereas the T time was adjusted between the 6th and 16th day (mean 11th). Mean ISS score was 22.59 (9 to 50). Classification according to trauma type was TBI (*n *= 44) followed by thoracic trauma. Thirty-one male survivors were discharged from the ICU, to the ward. The mortality rate amounts to 47 cases due to infectious/non-infectious nosocomial complications and multiorgan dysfunction syndrome. Clinical ISS, the type of injury, ICU length of stay (LOS), T day, demographic (gender, age) data and ICU outcome were registered. Statistical analysis was performed with GraphPad 5.0.

## Results

There is positive significant correlation between T day and LOS of injured patients (*P *< 0.001, Spearman coefficient = 0.1672). Statistical analysis by Mann-Whitney test, between groups A and B, showed significant differences in ICU LOS (*P *< 0.001); no significant differences (*P *< 0.05) were found for age, ISS and outcome (Table 1).

**Table 1:** ICU data

					Outcome	
	
ICU data	ISS	Age	ICU LOS (days)	Tracheostomy day	Survival	Mortality
A <10	16 ± 87 (16/45)	66 ± 94 (28/87)	602 17.2 ± 21.5 (4/47)	5.5 ± 6.5 (1/10)	*n *= 13 (43.3%)	*n *= 17 (57.7%)
B >10	16 ± 25 (9/50)	64 ± 87 (23/93)	1,496 25 ± 34 (9/95)	15 ± 26 (11/23)	*n *= 18 (40%)	*n *= 27 (60%)
*t *test	NS	NS	<0.001	<0.001	NS	NS

Data presented as median ± IQR.

## Conclusion

The optimum and early time point of tracheostomy seems to be directly related with LOS in the ICU, independently of the rate of ISS, patient's age and outcome. These results could account for ICU cost-effectiveness, as diminished LOS decreased the overall cost.
